# Case report: Torsade de pointes induced by the bigeminy result from retrograde ventriculoatrial activation in VVI pacing and resolved by intentional atrial pacing

**DOI:** 10.3389/fcvm.2023.1156658

**Published:** 2023-05-24

**Authors:** Song-Yun Chu, Qin-Hui Sheng, Qiu-Ping Shi, Lin Qiu, Lin Wu, Jing Zhou

**Affiliations:** Department of Cardiology, Peking University First Hospital, Beijing, China

**Keywords:** long QT syndrome (LQTS), torsade de pointes (TdP), pacemaker, implantable cardioversion defibrillator, atrioventricular sequential pacing

## Abstract

**Introduction:**

While pacing has been used for long QT syndrome (LQTs), the optimal pacing modality is controversial.

**Case:**

We report a woman with bradycardia and a recently implanted single-chamber pacemaker experienced multiple syncope. No device dysfunction was found. Multiple Torsade de Pointes (TdP) induced by the bigeminy result from retrograde ventriculoatrial (VA) activation in VVI pacing were demonstrated in the scenario of previously unidentified LQTs. Replacement for a dual-chamber ICD and intentional atrial pacing eliminated the VA conduction and symptoms.

**Conclusion:**

Pacing without atrioventricular sequence might be catastrophic in LQTs. Atrial pacing and atrioventricular synchrony should be highlighted.

## 1. Introduction

Pacemakers have been routinely used for treating bradycardia. For patients with long QT syndrome (LQTs), the pacing is also helpful for those who remain symptomatic despite being on beta-blockers, especially when bradycardia facilitates TdP (1, 2). While guidelines have established recommendations for permanent pacing in patients with LQTS and suggest ICD therapy for patients with risk factors for sudden cardiac death, formal recommendations for optimal device choice and programming for LQTS are not available.

## 2. Case report

**Figure 1:**
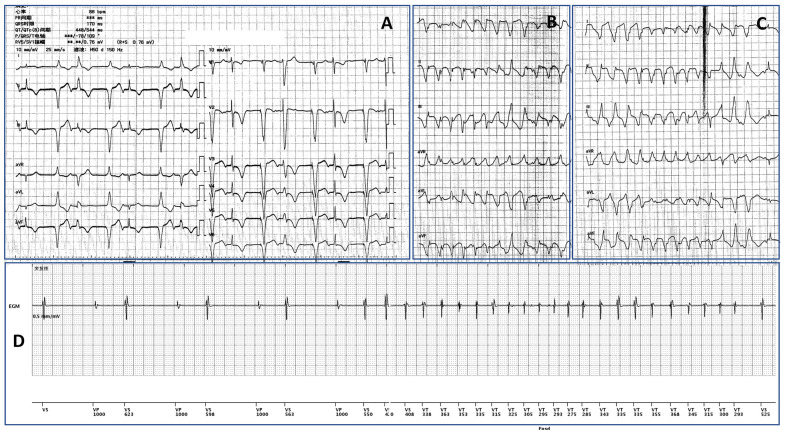
(**A**) VVI pacing led to repetitive VA retrograde conduction and the subsequent atrial activation conducted to capture the ventricle. The bigeminy led to short-long cycles and resulted in multiple Torsade de Pointes (TdP) shown in (**B and C**). (**D**) The EGM recorded the onset of one episode of the TdP, indicating the sequential relationship between the bigeminy and the malignant arrhythmia.

We reported a case of a 62-year-old woman who was admitted for intermittent dizziness and syncope. One month earlier, the patient received single-chamber pacemaker implantation for significant fatigue due to sinus bradycardia and asystole at a local hospital. No improvement in her symptoms was noted, and multiple syncope attacks prompted her visit. No dysfunction of the device was found during interrogation. However, telemetric ECG monitoring and EGM demonstrated Torsade de Pointes induced by the bigeminy result from repetitive retrograde ventriculoatrial (VA) activation in VVI pacing and sequential atrial impulses conducted to the ventricles through intact conduction systems (Figure 1).

**Figure 2:**
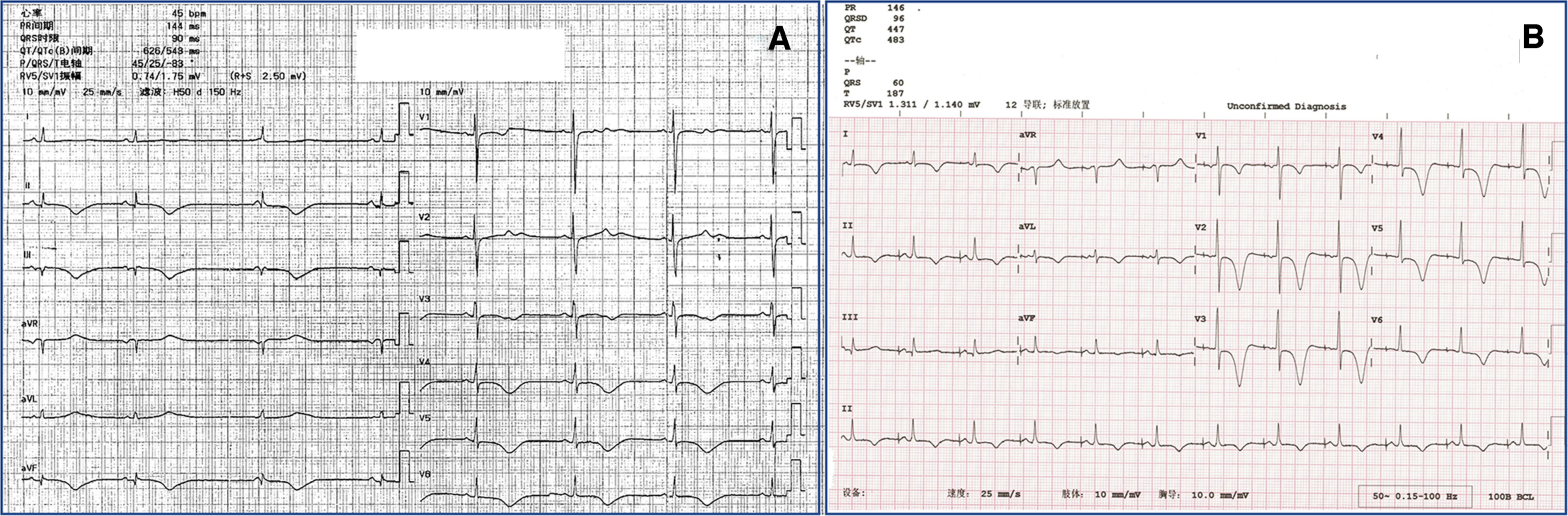
(**A**) Baseline 12-lead ECG of the patient before the single-chamber pacemaker implantation showed significant sinus bradycardia, junctional escape rhythm, and long QTc of 543 ms. (**B**) Replacement for a dual-chamber implantable cardiac defibrillator and intentionally higher atrial base rate of 70 bpm resulted in eliminating VA retrograde conduction and shortened QTc of 483 ms.

In the scenario of previously unidentified LQTs (Figure 2A), the bigeminy pattern created short-long cycles which contributed to the malignant arrhythmia attacks. Electrolyte disturbances and coronary artery disease as the reversible cause of the LQTs were ruled out, and a beta-blocker was initiated. Replacement of the device for a dual-chamber implantable cardiac defibrillator (ICD) and intentional atrial pacing eliminated the VA retrograde activation. Also, the higher base rate of 70 bpm helps to shorten the QTc (Figure 2B). In the follow-up, the patient reported significant improvement in her life quality with no syncope recurrence. No malignant arrhythmia was recorded by the ICD either. One missense variant in α-1,2-glucosyltransferase (ALG10c.1247G > A; p.Arg416His) of the patient was identified by whole-exome sequencing, which was furtherly verified by Sanger sequencing.

## 3. Discussion

Our case suggests the pivotal clinical significance of understanding the whole picture of an arrhythmia syndrome in decision-making. Although pacing is suggested as one of the treatment measures for LQTs, inappropriate device and setting might lead to unwanted outcomes.

The patient's story began with the implantation of a single-chamber pacemaker for sinus bradycardia. In patients with sinus node dysfunction (SND), however, studies have found that dual-chamber AV sequential pacing was superior to single-chamber ventricular-based pacing in reducing the risk of atrial fibrillation, stroke, and pacemaker syndrome (3, 4). Potential exceptions are those with advanced age and/or frail patients with infrequent pauses who have limited functional capacity and/or a short life expectancy (2). The patient did not fall into the exceptional category, and a single-chamber device might not be appropriate.

Notably, pacemaker syndrome has been reported to occur in ∼25% of patients with SND in VVI pacing (5, 6) and is associated with a reduced quality of life (6, 7). The patient had shown the predisposing characteristic for pacemaker syndrome with spontaneous VA retrograde conduction (8). It is natural to speculate that pacemaker syndrome was the culprit of the exacerbation of the symptoms. The base rate of the pacemaker was once set to 45 bpm at the local hospital to decrease the retrograde conduction. With no clinical improvement, insufficient correction of the bradycardia was suspected instead, and the base rate was changed to 70 bpm. The alteration could not alleviate the symptoms either. In fact, a higher programmed base rate was associated with the development of pacemaker syndrome, and a higher percentage of paced beats was an independent predictor (6).

When we continued to focus on the pre-assumption of pacemaker syndrome, findings in EGM and concurrent symptoms and telemetry suggested Tdp as the cause of the patient's loss of consciousness. With no prior cardiac arrest attack or associated family history, the prolonged QTc in the 12-lead ECG was attributed to the significant bradycardia and neglected. The bigeminy resulting from repetitive retrograde VA activation in VVI pacing created the short-long cycles contributing to the increased repolarization dispersion and enhanced early after-depolarizations. Both mechanisms are key to arrhythmia initiation in LQTs. Furthermore, the missense mutant of ALG10 identified in this case exhibits sequence homology with KCR1, therefore, might modulate IKr sensitivity (9). The multi-hit model (10), with the mutation serving as a genetic predisposition and bigeminy as the trigger, might result in the occurrence of Tdp.

ICD combined pacing is considered reasonable in patients with LQTS (7, 8) while no specific recommendations for the pacing modalities and programming were suggested in the guidelines. Meanwhile, pacing at an increased rate has been proposed as an essential treatment in LQTS because the modality shortens QT interval and the window of vulnerability for reinduction of Tdp (2). In our case, though, rapid ventricular pacing did not help if it did not accelerate the syncope attacks. It is not only because this pacing mode can cause heterogeneous ventricular depolarization that can be proarrhythmic (7), but also because the increased bigeminies and short-long couplings could further strengthen the vicious cycle.

The problem seemingly comes down to eliminating the retrograde VA activation as an initiation factor for both potential pacemaker syndrome and Tdp. Slow pathway ablation was an apparent approach. However, the AV desynchrony would not be solved as the sinus arrest and irregularity persists when only ventricular sensing and pacing exist. Also, ablation at the AV junction would have limited influence on the ventricular repolarization process, which defines the QT interval. On the other hand, increased-rate atrial pacing has been reported to shorten the QTc and reduce the risk of recurrent arrhythmia breakthroughs (7, 11, 12). Traditional right ventricular pacing, as shown in our case, should be minimized. The novel conduction system pacing (CSP) would be a promising modality for physiological ventricular activation in patients who need ventricular pacing (13, 14), although the sole adoption of CSP would not solve VA retrograde conduction. For our patient with sinus node dysfunction instead of atrioventricular block (AVB), dual-chamber ICD with minimized ventricular pacing algorism by using defibrillation RV lead implanted on the septum would be an optimal choice. Indeed, the intentional atrial pacing at 70 pm served as overdrive and effectively prevented the AV desynchrony. QTc was also significantly shortened.

## 4. Conclusion

Loss of atrioventricular sequence in LQTs might be catastrophic. Atrial pacing and atrioventricular synchrony should be highlighted as pacing strategies for LQTs.

## 5. Clinical perspective

As in our case, patients with channelopathies often act as candidates for cardiac implantable electronic devices. ICD has often been recommended for the prevention of sudden cardiac death in this population (15, 16). Consensus on the need for pacing and/or the optimal device selection, though, has not been obtained in most current guidelines. For instance, high-risk AVB has been reported in Brugada syndrome, especially in patients with baseline long PR interval (16). Considering this, a transvenous ICD with pacing capabilities should be considered instead of a simplified subcutaneous ICD. Future studies will be necessary to provide more evidence and recommendation on the tailored devices selection strategy.

## Data Availability

The original contributions presented in the study are included in the article, further inquiries can be directed to the corresponding author.
